# Spectral Dynamics of Resting State fMRI Within the Ventral Tegmental Area and Dorsal Raphe Nuclei in Medication-Free Major Depressive Disorder in Young Adults

**DOI:** 10.3389/fpsyt.2018.00163

**Published:** 2018-05-11

**Authors:** Afra Wohlschläger, Harish Karne, Denis Jordan, Mark J. Lowe, Stephen E. Jones, Amit Anand

**Affiliations:** ^1^Department of Diagonistic and Interventional Neuroradiology and TUMNIC, Technische Universität München, Munich, Germany; ^2^Center for Behavioral Health, Cleveland Clinic, Cleveland, OH, United States; ^3^Department of Anesthesiology, Klinikum Rechts der Isar, Technische Universität München, Munich, Germany; ^4^Radiology Institute, Cleveland Clinic, Cleveland, OH, United States

**Keywords:** dorsal raphe nucleus, ventral tegmental nucleus, resting state fMRI, resting state, serotonin, dopamine, depression

## Abstract

**Background:** Dorsal raphe nucleus (DRN) and ventral tegmental area (VTA) are major brainstem monamine nuclei consisting of serotonin and dopamine neurons respectively. Animal studies show that firing patterns in both nuclei are altered when animals exhibit depression like behaviors. Functional MRI studies in humans have shown reduced VTA activation and DRN connectivity in depression. This study for the first time aims at investigating the functional integrity of local neuronal firing concurrently in both the VTA and DRN *in vivo* in humans using spectral analysis of resting state low frequency fluctuation fMRI.

**Method:** A total of 97 medication-free subjects—67 medication-free young patients (ages 18–30) with major depressive disorder and 30 closely matched healthy controls were included in the study to detect aberrant dynamics in DRN and VTA. For the investigation of altered localized dynamics we conducted power spectral analysis and above this spectral cross correlation between the two groups. Complementary to this, spectral dependence of permutation entropy, an information theoretical measure, was compared between groups.

**Results:** Patients displayed significant spectral slowing in VTA vs. controls (*p* = 0.035, corrected). In DRN, spectral slowing was less pronounced, but the amount of slowing significantly correlated with 17-item Hamilton Depression Rating scores of depression severity (*p* = 0.038). Signal complexity as assessed via permutation entropy showed spectral alterations inline with the results on spectral slowing.

**Conclusion:** Our results indicate that altered functional dynamics of VTA and DRN in depression can be detected from regional fMRI signal. On this basis, impact of antidepressant treatment and treatment response can be assessed using these markers in future studies.

## Introduction

The three major Brain stem monoamine nuclei (BSMN)—ventral tegmental area (VTA), dorsal raphe nucleus (DRN), and locus coeruleus (LC) consist of clusters of dopaminergic (DA), serotonergic (5-HT) and noradrenergic (NE) producing neurons respectively. Though they consist of a few thousand of neurons their broad projections to nearly all cortico-limbic regions have significant modulatory effects on brain regions and circuits. The involvement of the monoamine in reward, arousal, vegetative and cognitive functions is well established. Depression is frequently associated with abnormalities in these functions and therefore monoamine dysfunction has been investigated in detail in animal and human models of depression (1, 2). Depletion of monoamines leads to development of depression like symptoms and reverses the therapeutic effects of antidepressants (3). Most antidepressants act on monoamine reuptake mechanisms or monoamine post-synaptic receptors (4). Despite the generally accepted critical role that the BSMN play in the pathophysiology of depression the functional integrity of BSMN pathways have been less well studied. This study is focused on the study of VTA and DRN as LC is too small size to be studied with the methods used in this study.

The VTA is located in the midbrain and primarily consists of dopaminergic neurons with mesocortical and mesolimbic pathways project to cortical and limbic areas respectively (5). The mesolimbic pathway, a part of the medial forebrain bundle (MFB), connects to the limbic areas, particularly the NAcc and constitutes a major reward activated pathway and has been implicated in addictions as well as anhedonia frequently seen in depression (6). Importantly, in recent studies of deep brain stimulation (DBS) for treatment of depression direct or indirect stimulation of the VTA has been hypothesized (7, 8). Lewy bodies in the VTA have been associated with significant depressive symptoms (9). Therefore, regional functional abnormalities are likely to be present in depression.

The DRN is the largest of the raphe nuclei is responsible for majority of the 5-HT transmission to the brain. It is located in the midbrain in the ventral part of the periaqueductal gray matter in the midline. Its rostral end is at the level of the oculomotor nucleus and its caudal subdivision reaches well into the periventricular gray matter of the rostral pons. It primarily consists of serotonergic neurons though other neurotransmitters are also present. Through its ventral ascending pathways, it innervates many limbic and cortical areas involved in mood regulation such as amygdala and the anterior cingulate cortex (ACC). One study reported a 31% decrease in neurons in depressed patients (10) though another study did not (11). Post-mortem studies have reported 5HT1A receptor and tryptophan hydroxylase abnormalities in DRN in depressed suicide subjects (12, 13). Single photon emission computerized imaging (SPECT) and positron emission tomography (PET) studies have reported 5-HT transporter uptake abnormality in the midbrain (14) as well as prediction of antidepressant response based on the mid brain 5-HTT uptake (15, 16). PET scan studies have reported reduction in 5HT1A binding after SSRI treatment (17). Furthermore, baseline elevated 5HT1A binding in the raphe nucleus was reported to be a predictor of treatment response while binding in the cortical and subcortical regions was not (18). Functional connectivity of the DRN has been reported to be decreased when symptoms of depression are mimicked by tryptophan depletion (19).

Several studies prove that DRN as well as VTA possess distinct BOLD representations which allow for detecting characteristic connectivity patterns in the brain (DRN: (20), VTA: (21)). Changed integration of DRN and VTA into overall brain orchestration in psychiatric diseases is observable via fMRI, like in ADHD (22) and in affective disorders (23, 24).

Until now functional integrity of VTA and DRN has been difficult to study because of lack of an appropriate localized metric. In resting-state fMRI, in the absence of an external paradigm, regional signal can be inspected for power spectral differences, differences in spectral cross-correlation, or differences in information content using entropy measures. Both VTA and DRN nuclei show pathological alterations in their firing patterns in MDD in rodent studies (25–29). In VTA, simulations show that timing relations between tonic and phasic firing directly relate to D1 vs. D2 receptor occupancy (30) and therefore seems to be an integral part of the disease mechanism. Therefore, spectral changes can be used to measure changes in the functional integrity of these nuclei. Appearance of bursts in phasic firing of the mesolimbic dopamine system has been shown to occur down to a frequency range of 0.1–0.15 Hz (31–33). This range falls well into the domain which might be observable by fMRI (0.01–0.17 Hz) (34, 35). Also, a small portion of serotonergic neurons in DRN seem to exhibit slow frequency activity (36, 37). Although, BOLD signal from fMRI cannot provide information on precise neuronal timing, changes in the slow regime accessible by BOLD fMRI are indicative of altered neuronal dynamics on a quicker scale.

**Figure 1:**
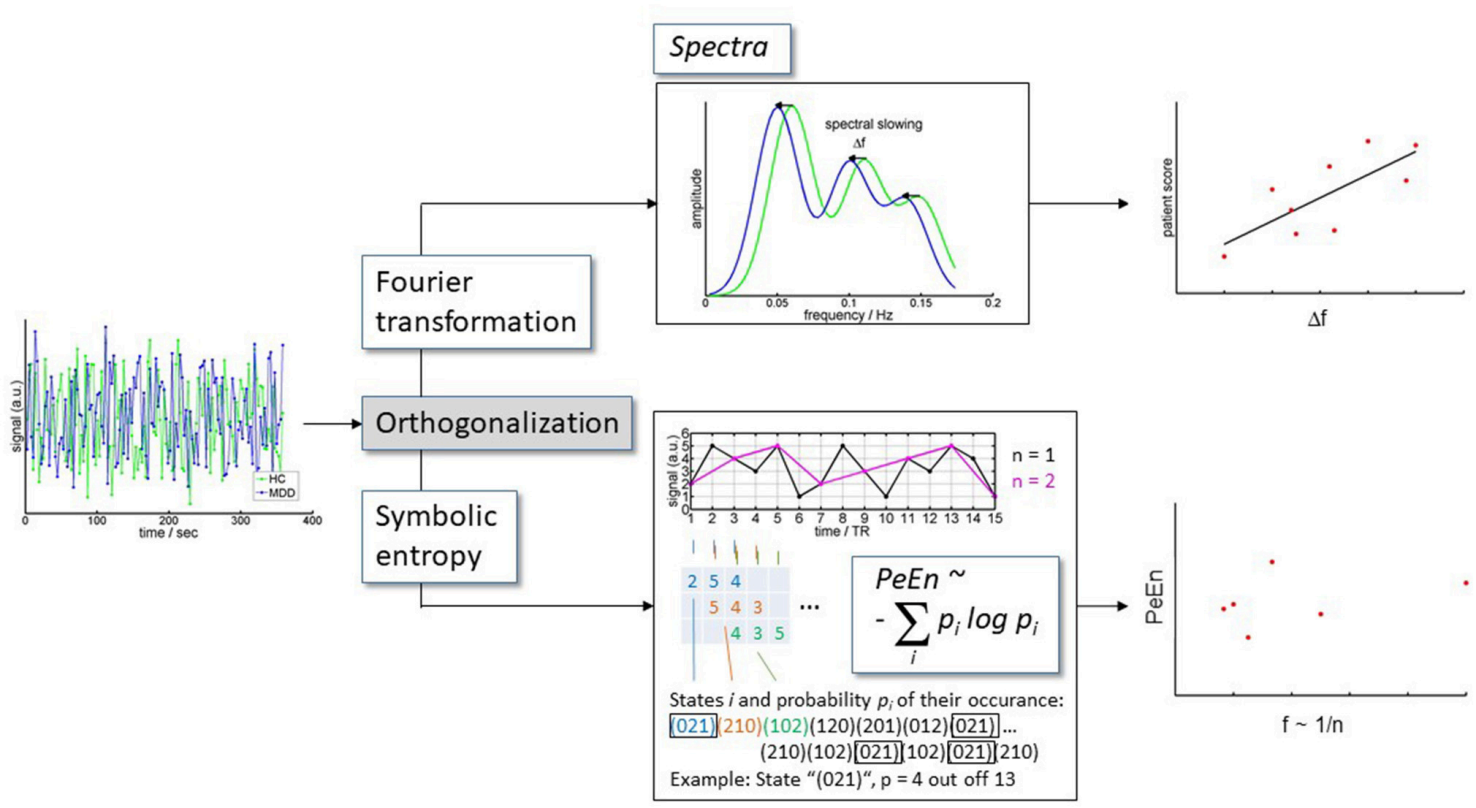
Analysis scheme. BOLD time series extracted from DRN and VTA were orthogonalized toward each other for each subject. Subsequently, the orthogonalized time courses were (i) converted to spectra, and (ii) were analyzed for information content.

Furthermore, shifts in spectral cross correlation i.e., correlation of the amplitude of frequency bands of resting state low frequency BOLD fluctuations (rLFBF) between groups allows for the examination of differences in dynamic signal properties within regions of interest. Similarly, a complementary approach of permutation entropy, analyzes the signal complexity at certain frequency bands explicitly ignoring absolute spectral power values (overview in Figure 1).

We hypothesized that pathological malfunctioning of DRN and VTA has detectable correlates in their BOLD fMRI representations. We therefore aimed at understanding the representation of DRN and VTA via rs-fmri in terms of time course variance, frequency spectra, and spectral information content. We hypothesized that in depression spectral slowing of the BOLD signal will be present in the VTA and DRN and that the degree of slowing will correlate with severity of depressive symptoms.

## Methods

### Participants

Subjects for this study were derived from a study of depression in young adults (Age 18–30). Subjects were recruited from the outpatient clinic at Cleveland Clinic, Center for Behavioral Health and by advertisement from the community. A total of 91 depressed and 39 healthy controls were included in the study. Out of the 91 depressed subjects some subjects were removed for the following reasons: 5 subjects did not do the scan, 6 subjects due to motion, 5 subjects slept during the scan, 7 subjects for processing and image quality, 1 subject was not used due to other reason. Out of the 39 healthy subjects were removed for the following reasons 2 subjects did not do the scan, 1 subject due to motion, 2 subjects for family history, 3 for image quality and processing, 1 for sleep. Therefore, data from 97 subjects—67 MDD and 30 HC was included in the analysis. This young age group (18–30 years) population was thought to be ideal to study physiological aspects of depression as confound of age related effects can be minimized. All subjects took part in the study after signing an informed consent form approved by the Investigational Review Board (IRB) at Cleveland Clinic Foundation. Both patients and HC were paid $25 for screening and $75 for MRI scan. All subjects underwent a detailed structured diagnostic interview—Mini Neuropsychiatric Interview (MINI) that generated a DSM-IV diagnosis.

Inclusion criteria for patients were: Ages 15–30 years and ability to give voluntary informed consent; (1) Satisfy criteria for DSM-IV-TR Major Depressive Episode using a Structured Interview; (2) Never met criteria for mania or hypomania; (3) 17-item Hamilton Depression Rating Scale score (HDRS) > 18 and < 25; (4) Young Mania Rating Scale (YMRS) score < 10; (5) Satisfy safety criteria to undergo an MRI scan; (6) Able to be managed as outpatients during the study as ascertained by the following—(i) Clinical Global Severity Scale < 5 i.e., moderately ill, (ii) No significant suicidal or homicidal ideation or grossly disabled. Exclusion criteria for all patients were: (1) meeting DSM-IV criteria for schizophrenia, schizoaffective disorder, or an anxiety disorder as a primary diagnosis; (2) use of psychotropics in the past 2 weeks; use of fluoxetine in the past 5 weeks; (3) acutely suicidal or homicidal or requiring inpatient treatment; (4) meeting DSM-IV criteria for substance dependence within the past year, except caffeine or nicotine; (5) positive urinary toxicology screening at baseline; (6) use of alcohol in the past 1 week; serious medical or neurological illness; (7) current pregnancy or breast feeding; (8) metallic implants or other contraindications to MRI.

Inclusion criteria for healthy subjects were: (1) ages 18–30 years and ability to give voluntary informed consent; (2) no history of psychiatric illness or substance abuse or dependence; (3) no significant family history of psychiatric or neurological illness; (4) not currently taking any prescription or centrally acting medications; (5) no use of alcohol in the past 1 week; and no serious medical or neurological illness. Exclusion criteria for healthy subjects were: (1) pregnant or breast-feeding and (2) metallic implants or other contraindication to MRI.

### Functional MRI acquisition

Imaging data consists of T1- and T2-weighted structural scans and resting state fMRI (RS-fMRI). All imaging data was acquired at the Cleveland Clinic Main Campus imaging center using a Siemens 3T Trio and Prisma MR Scanner (Siemens AG, Berlin, Germany) with a 32 receive channel head coil (Nova Medical, Inc., Wilmington, MA) and electronically transferred to the Cleveland Clinic imaging archive system.

Anatomic scans: T1-weighted structural images were acquired with a MPRAGE sequence (echo time = 2.98 ms, repetition time = 2,300 ms, inversion time = 900 ms, flip angle = 9°, field of view = 240 × 256 mm, slice thickness = 1.2 mm).

RS-fMRI scans: 6:16 min scan with eyes open looking at a fixation cross. 39 3.5 mm-thick slices, 2.5 × 2.5 mm voxels in-plane resolution, TR/TE = 2,800/29 ms.

All participants were fitted for a bite bar to restrict head motion during scanning. All EPI data was corrected for spatial distortion using a fieldmap-based shiftmap before further analysis. This step allows matching anatomy to warped regions of brain, including the amygdala and OFC. For resting state functional connectivity, 132 image volumes were acquired in 6:16 min. The first 4 volumes were discarded and data was analyzed on the resulting 128 volumes. Functional connectivity image volumes were acquired while subjects were in the resting state with eyes open looking at a fixation cross, instructed to think nothing in particular. After the resting state scan was completed, the subjects were asked whether they stayed awake and complied with instructions and only those who reported complying with the instructions were included in the analysis.

### Data analyses

#### Image analysis

##### Pre-processing including motion correction

The images were corrected for physiologic noise (38–40) using signals obtained with PESTICA (Physiologic Estimation by Temporal ICA) (41). Special attention was paid to motion correction because both linear and non-linear motion artifacts have been shown to affect functional result (42, 43). Motion correction was performed using SLice-Oriented MOtion COrrection (SLOMOCO) (39). SLOMOCO first performs an in-plane slicewise motion registration followed by an out-of-plane motion parameter estimation and regularization. The regularized out-of-plane and residual in-plane motion parameters are used in a slice-specific second-order motion model that accounts for the effect of adjacent slice motion into or out of the slice of interest as well as the present slice. Finally, the software regresses the physiologic noise model in parallel with the slice-wise second-order motion model, and this regression correction comprises the last stage of SLOMOCO to produce data that has been corrected for physiologic noise and motion.

After motion correction, images were corrected for non-neural sources of variance using a regression-based correction with time series obtained from eroded white matter and ventricular mask (44). The corrected images were normalized to Montreal Neurological Institute (MNI) space, resampled to 2 mm isotropic voxels and finally, bandpass filtered to retain low-frequency fluctuations [0.008–0.175 Hz (upper limit of sampling bandwidth)] using 3dBandpass, from AFNI (Analysis of Functional Neuroimages) (45). For every scan the number of motion-corrupted volumes was identified using the Jiang average voxel displacement measurement (46) computed from the slice-wise motion parameters from SLOMOCO. A corrupted volume was defined as a volume where at least one slice within that volume experienced greater than 1 mm of out of plane motion. Any subject with 13 or more volumes (i.e., more than 10% of volumes) with greater than 1 mm of out of plane motion were excluded from the analysis (39, 46).

##### Derivation of ROIs for DRN and VTA

Due to limited contrast resolution of small brainstem nuclei in brain MRIs and the lack of a standard corresponding MRI atlas, regions of interest (ROI) were identified using operationalized anatomical criteria developed by an experienced neuroradiologist (S.E.J) for the brainstem based on two well-established brainstem atlases (47 and Duvernoy's Atlas of the Human Brainstem and Cerebellum: High-Field MRI, Surface Anatomy, Internal Structure, Vascularization, and 3D Sectional Anatomy, 2009) (47, 48) for defining the DRN and VTA ROIs.

The procedure has three steps: (1) Define a brainstem axis that will form the perpendicular to axial planes; (2) Defining the superior and inferior extents of the DRN and VTA; (3) Manually drawing the extents of the DRN and VTA in the axial plane. The brainstem axis and axial planes are defined as perpendicular to the mid-sagittal plane and parallel to the line between the central mammillary bodies and the intercollicular fossa. The Paxinos atlas is used to determine the superior and inferior extents because of its uniform axial spacing and well-marked measured longitudinal reference of the axial image with respect to the obex (Paxinos, Figures 50–59) (47). For the DRN, we define the inferior extent as the plane containing the inferior edge of the inferior colliculus (IC) and the superior extent as the plane halfway between the central superior colliculus (SC) and the intercollicular fossa—a superior-inferior distance of typically 7 mm. For the VTA we define the inferior extent as the plane containing the center of the IC and the most superior extent as the plane continuing the center of the superior colliculus (SC)—a superior-inferior distance of typically 6 mm. We use the Duverney atlas to draw the DRN and VTA ROIs in the axial plane, as the Paxinos atlas has more detail than can be seen in the MRIs (Duverney chapter 4, page 55; and Figures 2.18–2.20) (48). In each axial slice, the midline periaquaductal gray matter (PaqGM) is marked off as a strip 1 mm anterior to the cerebral aqueduct. Extending anteriorly from the PaqGM toward the interpeduncular fossa (IpF), all the voxels on either side of the midline are split between the DRN and VTA. For the superior two thirds of DRN axial slices, the posterior two thirds of midline voxels between the IpF and PAq are DRN. For the inferior one third of DRN slices, the posterior half of midline voxels are DRN. Conversely, for the superior two thirds of VTA axial slices, the anterior one third of voxels between the IpF and PAq is VTA. For the inferior one third, the anterior half of midline voxel is VTA. Regarding lateral extent of the DRN, for the inferior two thirds of DRN axial slices a second line of DRN voxels are laterally added to the para-midline voxels from the most posterior DRN voxel to one third (anteriorly) up the midline strip of DRN voxels. Regarding lateral extent of the VTA, in all axial slices the VTA extends diagonally along the IpF border. The most superior slice extends 1 voxel, the next extends 2 voxels, and the remaining slices extend 3 voxels. A second row of VTA voxels adjoins lateral and parallel to the first diagonal along the IpF border for the inferior two thirds of axial VTA slices.

##### Region of interest templates and time course extraction

DRN & VTA ROI templates were constructed as described above individually for each of 17 healthy subjects. The overlapping areas from these 17 subjects was then used as the two templates for DRN and VTA respectively. These templates were then normalized to the MNI space using Statistical Parametric Mapping (SPM) version 12 software and then the normalized ROIs were used to extract resting state time series from all subjects.

##### Analysis of variance and time course characteristics

Time courses were analyzed in terms of their variance across time quantified by their standard deviations. Additionally, intra-subject correlations of the time courses between the two regions of interest were analyzed.

##### Analysis of frequency spectra (FFT and clipping)

Signal time courses for each subject were Fourier transformed using matlab (www.mathworks.com). This resulted in a frequency resolution of 2.8 ^*^ 10^−3^ Hz. Significance of group differences in spectral amplitudes were assessed via Wilcoxon rank sum test.

**Figure 2:**
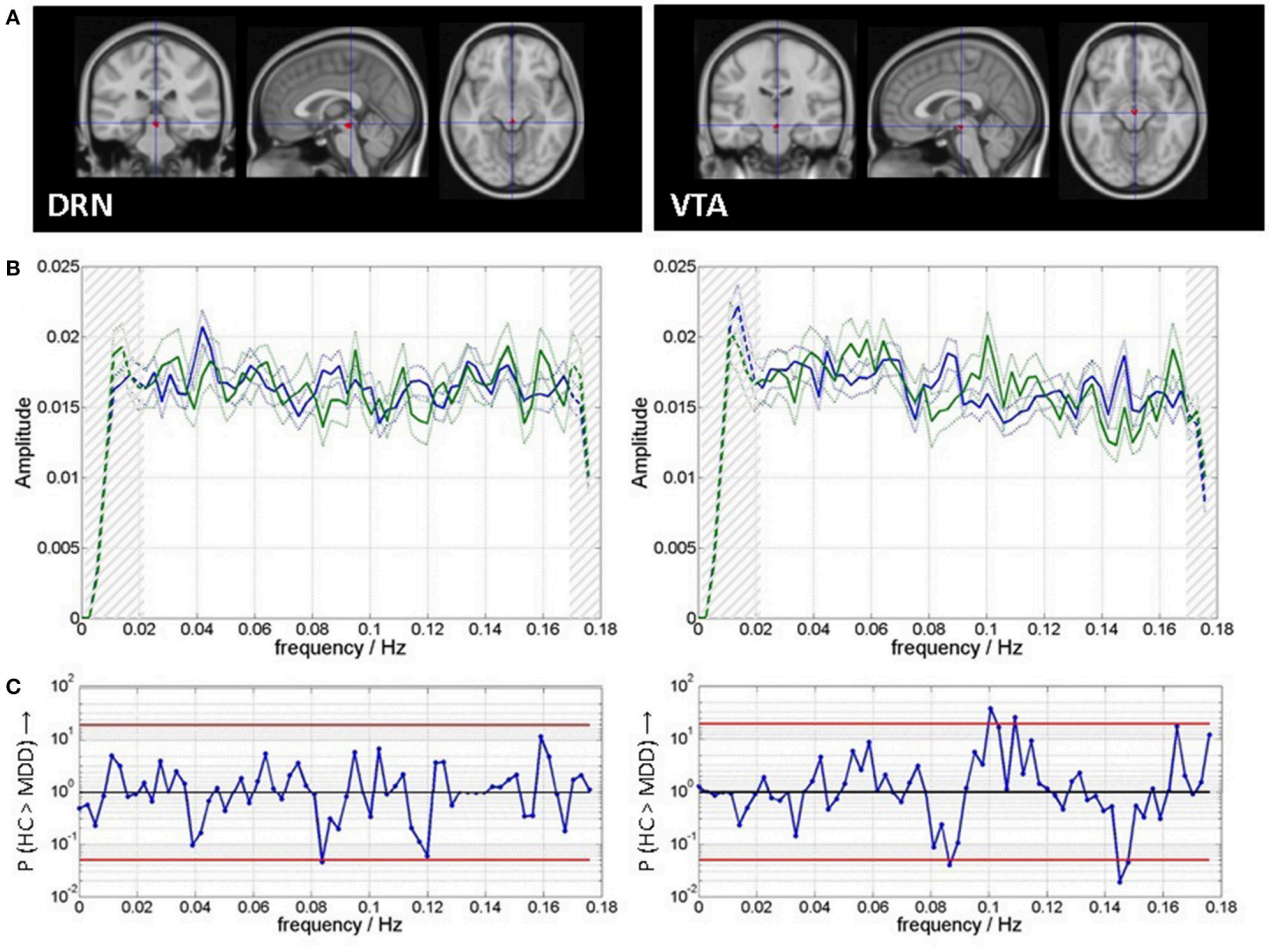
**(A)** Regions of interest for time course extraction and **(B)** mean and standard error of mean of spectra for patients (blue) and healthy controls (green). Clipping of spectra in subsequent analyses is indicated by hatching on both ends of the spectra. **(C)** Spectral group differences are indicated as *P*-values from Wilcoxon rank sum tests the red lines indicate *P* = 0.05, uncorrected for multiple comparisons.

In the spectra we did not find a 1/f decay of power with frequency f. Efficient noise removal and head motion correction might have led to minimizing the scale free properties, which are partly attributed to noise effects (49). Spectral cross correlation could be directly performed without any normalization procedure. The fact that spectra were flat across frequencies may be due to maximally reduced movement of the subjects, which could have helped in isolating unblurred signal from the ROIs of DRN and VTA. Spectral slowing in the patients was assessed by calculating the correlation between the spectra of each patient shifted toward the spectra of each of the 30 controls (Figure 2). Shifts by 1 to 8 resolution units in both directions (toward higher and lower frequencies) were included as well as zero shift, resulting in 17 different shifts. Pearson *R*-values were then Fisher-Z transformed to normal distribution and averaged per patient. This resulted in one value per patient. An identical procedure was performed for each control subject toward each of the other control subjects. Significance of these (Fisher-Z transformed) average correlation coefficients was then tested by Wilcoxon signed rank test toward zero correlation. In order to rule out effects of the signal filter on the spectra, 8 data points (0.0223 Hz) and 4 data points (0.0112 Hz) at the lower and upper limits, respectively, of the spectra were excluded from all subsequent analyses (see Figure 2, not from the figures). A spectral range of (0.0223–0.1646) Hz was therefore analyzed.

For each individual patient, the shift with peak correlation was determined per region of interest (Figure 2). This shift was then correlated to the clinical score of that patient (HamD) across patients per region. Regressors coding for scanner type, slice-wise mean motion, volume-wise mean motion, average cardiac rate, and variance of cardiac rate were included as nuisance regressors into the analysis, in order to find effects over and above independent variance sources.

##### Spectral information content [permutation entropy (PeEn)]

PeEn analyzes amplitude orders instead of absolute amplitudes. PeEn quantifies the regularity structure in these orders of the signal values in a time series as measure of signal complexity or “information content” (50). The basic strategy of PeEn calculation is indicated in Figure 1. PeEn therefore is non-parametric and (a) is more robust against signal distortions, and poorly known characteristics of the underlying dynamics (51, 52), and (b) aims at complementary aspects to the analysis of spectral power based on signal amplitudes. The analysis requires the specification of so called embedding parameters. These are the dimension m, i.e., the number of time points in each time window used for assigning the order ranking, and the sampling rate. An embedding dimension *m* = 3 was used matching the duration of the canonical hemodynamic response function. Maximal accessible sampling rate equals the inverse of the TR. Variance on a slower time scale can be investigated by choosing the sampling rate as inverses of multiples n of TR and disregarding the respective values in between. We investigated n in the range of 1 to 7 roughly corresponding to a frequency range calculated by *f* = 1/(n ^*^ 2 ^*^ TR) of 0.026 to 0.179 Hz. We used the implementation in LabView as published for EEG data by Jordan et al. (52).

## Results

### Scanning parameters and physiological parameters

As can be seen from Table 1 motion parameters as well as physiological parameters did not differ systematically between groups.

**Table 1:** Group demographic, illness and scanning characteristics.

	**MDD**	**HC**	**Significance of group difference**
*N*	67	30	
**SUBJECT AND MEASUREMENT CHARACTERISTICS**			
Age (median [min – max])	24 [18–30]	24 [18–30]	
Gender (#-females)	46 (68%)	22 (73%)	
*Clinical parameters (median [min – max])*			*P (Wilkoxon rank sum test)*
Duration of current episode / weeks	15 [3–416]	N/A	
Number of depressive episodes	14 [1.5–144]	N/A	
Medication Free period / weeks (23 subjects were medication naïve)	42 [2–242]	N/A	
17-item Hamilton Depression Rating Scale (HAM-D)	17 [9–27]	0 [0–3]	<0.001
Young Mania Rating Scale (YMRS)	0 [0–11]	0 [0–0]	<0.001
*Movement parameters (mean ± SD)*			*P (Wilkoxon rank sum test)*
Slice-wise mean motion (mm)	0.256 ± 0.061	0.252 ± 0.061	0.73
Volume-wise mean motion (mm)	0.35 ± 0.11	0.37 ± 0.13	0.87
*Physiological parameters (mean ± SD)*			*P (Wilkoxon rank sum test)*
Average cardiac / BpM	65 ± 9	62 ± 6	0.31
Std cardiac / BpM	2 ± 3	2 ± 1	0.98

#### Descriptives of time courses

An analysis of the standard deviation the time course variance across the whole run of DRN and VTA was correlated across all subjects (Pearson correlation: *R* = 0.32, *p* = 0.002, *N* = 67). This means variance of one ROI was high in those subjects in which also that of the other ROI was high and vice versa. Variance was higher in DRN than in VTA across all subjects (*P* < 0.001, Wilcoxon rank sum test). Importantly, there were no group differences in magnitude of variance (p_DRN_ = 0.466, p_VTA_ = 0.376, *t*-tests).

Time courses between the two ROIs were highly correlated within subject as assessed on the Fisher-Z transformed Pearson correlation coefficients (*p* < 0.001, *t*-test). These correlations might be due to residual artifacts such as motion, although care was taken to minimize these effects. To exclude those common artifactual contributions, data from each of the ROIs were orthogonalized toward each other in the subsequent analyses in order to focus on region specific dynamics. There was no difference in the within subject interregional correlation between the two groups (*p* = 0.70, two-sample *t*-test).

In summary, the description of the time courses of VTA and DRN does not reveal any systematic differences between patient and control group. Orthogonalized time courses of the two ROIs were further investigated with regards to spectral differences and differences in information content.

#### Spectral differences and spectral slowing

Figure 2A shows the ROI templates used for extraction of time series. Figure 2B shows the spectra of VTA and DRN for both groups. Figure 2C shows that there are moderate between group differences significant at a level of *P*_u_ < 0.05 (Wilcoxon rank sum test, uncorrected for multiple comparisons): for DRN at 0.08 Hz (MDD > HC), and for VTA at 0.09 Hz (MDD > HC), at 0.10–0.11 Hz (HC > MDD), and at 0.15–0.18 Hz (MDD > HC).

**Figure 3:**
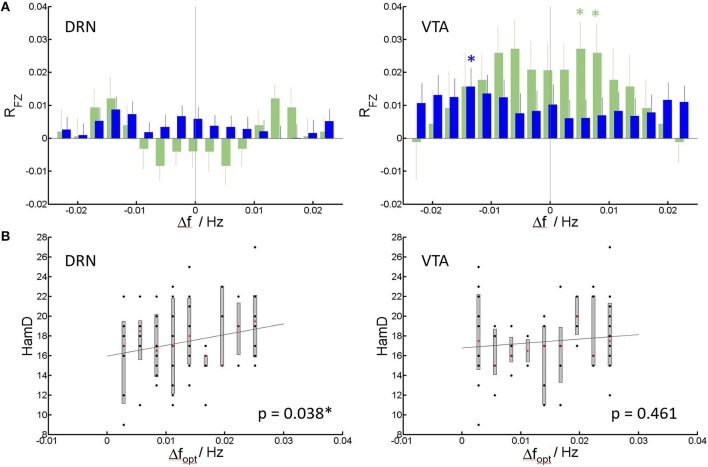
Spectral slowing. **(A)** Mean and standard error of mean of correlation coefficients RFZ (Fisher Z-transformed) of individual patients (blue) and controls (green) to the group of healthy controls (see Methods) vs. shift in frequency. Significant correlations at Pc < 0.05, corrected for multiple comparisons (Bonferroni-factor = 17) are indicated by an asterisk. **(B)** HamD score per patient vs. the frequency shift creating the highest (optimal) average correlation of patient spectra to spectra of healthy controls (Dfopt). Median, 75% confidence intervals and outliers are given for all realized shifts. Least square lines to the individual data are added as guide to the eye. Significance of the correlation (corrected for nuisance variables), is indicated within the panels.

Figure 3 shows the results of the spectral shift correlation analysis. In VTA the only significance in cross correlation occurs at a shift of 0.014 Hz (P_c_ = 0.035, Wilcoxon signed rank test, corrected for 17 tests on all shifts under concern) of patient' spectra toward healthy controls‘, i.e., patients spectra resemble those of healthy controls but at slower frequencies (Figure 3A). While the same analysis in healthy controls produces symmetric results, in MDD generally the correlation toward spectra of healthy controls improves when patient spectra are shifted toward higher frequencies, i.e., they are initially shifted toward lower frequencies. In DRN, no shift in cross-correlation showed significant correlation.

For each individual patient, the frequency shift to higher frequencies for optimal match to controls can be determined, and correlation to HamD can be calculated. For DRN, this shift positively correlates with the HamD scores of the individual patients (*p* = 0.038, Pearson correlation) (Figure 3B). This means higher HamD scores are associated to an increased spectral slowing in DRN. In VTA, there was no significant correlation (*p* = 0.461, Pearson correlation). Shift data were linearly corrected for nuisance regressors (see Methods section) prior to correlation to rule out possible effects of nuisances. The dependence is displayed in Figure 3C for uncorrected shift data (for clarity).

### Spectral information content

**Figure 4:**
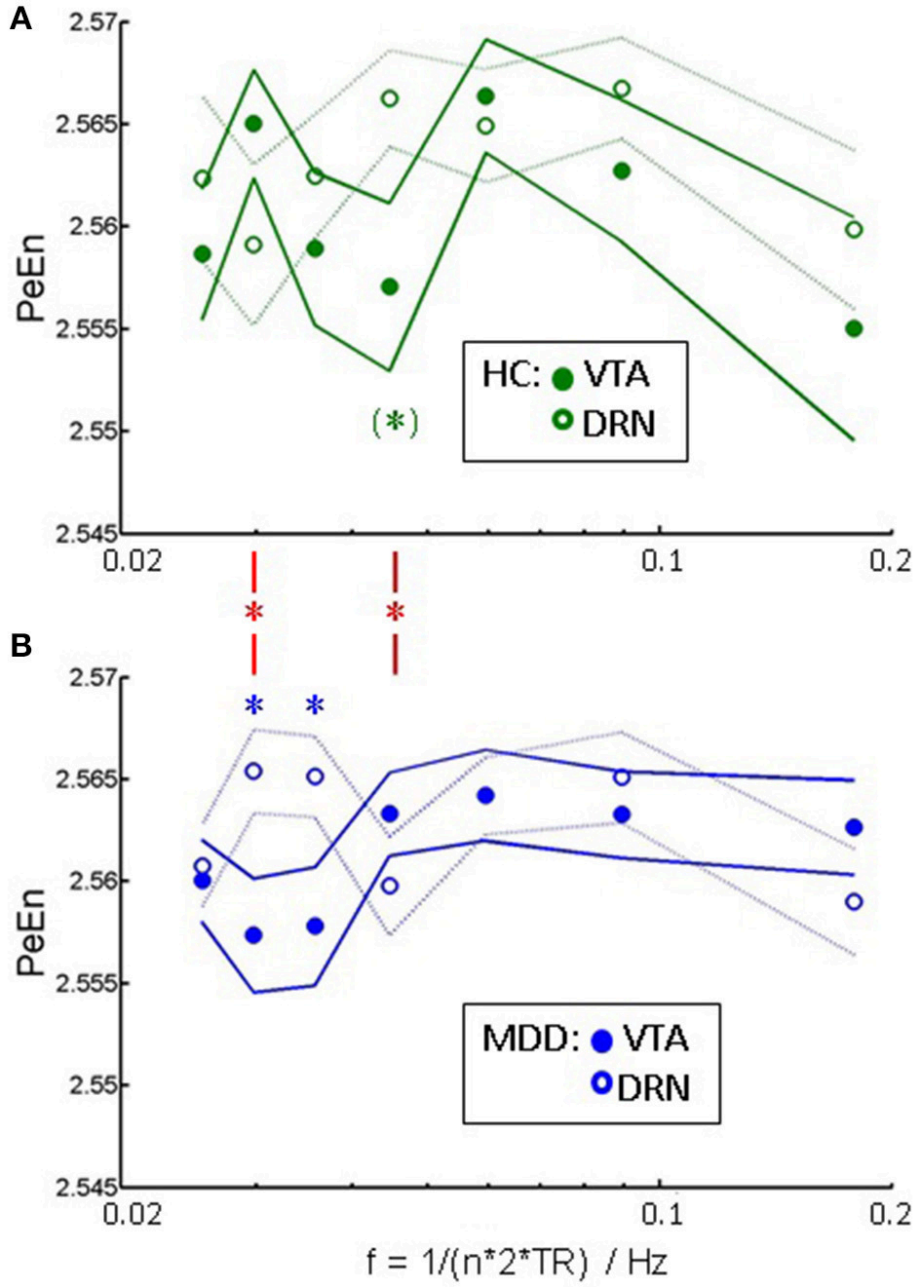
Signal complexity: PeEn vs. a correlate of frequency ~ 1/n in **(A)** healthy controls, and **(B)** patients as mean and standard error of mean. Significant within-group differences between the PeEn of the two ROIs at *P* < 0.05, uncorrected for multiple comparisons (Wilcoxon signed rank test) are indicated by asterisks. Red asterisks indicate significant interactions of group × ROI at the same significance level.

Figure 4 shows the information content as assessed via permutation entropy in DRN and VTA per group. Information content is within a comparable range across ROIs and groups. In healthy controls information content in DRN is, at trend, higher than in VTA at a frequency of about 0.045 Hz (P_u_ = 0.051, Wilcoxon signed rank test, uncorrected for multiple comparisons). In MDD, similarly information content is higher in DRN than in VTA, but at lower frequencies of 0.030 Hz (P_u_ = 0.028) and 0.036 Hz (P_u_ = 0.035). At two of those frequencies group x ROI interaction is significant (red asterisks) at 0.030 Hz (P_u_ = 0.017, Wilcoxon rank sum test on inter-regional differences), and at 0.045 Hz (P_u_ = 0.019).

## Discussion

In the present study, we analyzed the dynamics of resting state fMRI time courses from VTA and DRN and their pathological alterations in a group of young unmedicated MDD patients. We found a characteristic slowing of VTA and DRN dynamics in the frequency range accessible to fMRI. This slowing was significant but not dependent on clinical scores in VTA. In DRN the slowing was less pronounced but correlated with disease severity as assessed via the HamD score. Analysis of permutation entropy revealed an aberrant frequency distribution of signal complexity in MDD. Based on the underlying notion, that complexity is related to richness (or reduction) in information processing, the results on entropy strengthen a slowing of neuronal processing in VTA (trough in PeEn at lower frequencies) and DRN in MDD. Therefore, two independent measures, i.e., signal amplitude and signal complexity, provide a consistent picture.

As these abnormalities were seen in young adults with depression they are unlikely to be due to neurodgeneration associated with age of long-term effects of chronic depression. Several studies investigated the BOLD reflexion of DRN function (53, 54). Solomon and colleagues investigated tryptophan depletion, as model for the effect of low serotonin in the human brain (53). They reported pontine raphé enhancement in different frequency bands of BOLD signal upon tryptophane depletion. They found a significant increase in power in the ultralow frequency of below 0.033 Hz. A massive increase in the high frequencies of 0.125 to 0.25 Hz is in a range beyond the one accessible in this current study. Also, Weinstein et al. (54) show that raphé functional connectivity in acute tryptophan depletion changes selectively in different frequency bands. These findings highlight the ability of BOLD imaging to bridge the gap between system level functional connectivity investigations and analyses of biochemical effects in MDD.

VTA function is characterized by tonic and phasic activity. By its temporal characteristics, apart from larger scale feedback loops, candidates for BOLD visible activity could be envelopes of phasic burst train activity. These fall into the range 0.1–0.15 Hz (31–33). Although, generally evidence points at an increase in average phasic firing in MDD (25, 26, 55, 56), envelopes of phasic firing might be affected in a different way.

Generally, the hemodynamic response to the very diverse kinds of neuronal activity in brainstem nuclei is not known. It is assumed that metabolic demands elicit a BOLD response following the balloon model (57) and that variations to that time course are minor throughout the brain. Nevertheless, variable hemodynamic responses underlying the BOLD response are possible (58), so direct inference on any underlying neuronal dynamics can not be performed in a straight forward way. Although uncertainty about the exact shape of a hemodynamic response or folding effects due to low sampling rates in fMRI reduce precision of quantitative timing information, a change in BOLD dynamics is indicative of a change in underlying neuronal firing patterns.

Interpretation of BOLD spectral properties in context of inter-regional communication is established usually within a specific spectral range (54, 59). Deviating from these approaches, in the present study, we find a spectral shift rather than a lack in a specific spectral range. By its nature this rather points at reflecting regional alterations in neuronal firing.

The slowing of the spectra may be indicative of regional dysfunction. In the case of DRN this would relate to dysfunction of serotonergic neurons. Here we found that the slowing goes along with increased depression severity which is inline with the serotonergic dysfunction hypothesis of depression (60). Translational studies highlight the importance of establishing a link between transmitter physiology and BOLD representation to connect animal studies and human imaging studies especially focusing on MDD (61). Temporal dysbalance in depression toward slower rather than faster portions of the spectra both from fMRI as well as EEG have been discussed in context with altered experience of subjective time judgments in depression (62). Studies from electrophysiology and fMRI demonstrate that the brain actively exploits matched or nested oscillations for proper functioning (63). A selective slowing in central modules of brain function therefore must impact on interregional communication and information spread. Selective slowing of activity in major BSMN might be a linking piece between animal studies on transmitter systems, functional connectivity analysis in human and behavior.

Our findings of abnormalities in the VTA are consistent with that of other studies in depression where VTA abnormalities were found but not necessarily of the DRN. For example, Wilson and colleagues reported lower density tyrosine hydroxylase in the VTA in the postmortem brains of patients with late life depression but not in the DRN or locus coeruleus. VTA phasic firing has also been reported to be associated with development of depression in a social defeat paradigm in a mouse model. However, our findings also indicate that abnormalities of the DRN are more closely related to the severity of depressive symptoms underscoring the importance of sertonergic abnormalities. Together these findings support the hypothesis that sertonergic function may have a modulatory role on dopaminergic abnormalities and increase or decrease depression severity (2). This study is focused on the study of VTA and DRN as LC is too small size to be imaged with the methods used in this study. In future studies using higher resolution MRI techniques investigation of LC may also be possible and may give a better picture of the contribution of each of the BSMN in the pathophsophysiology of depression.

In conclusion we found systematic changes in the activity dynamics of the BSMN DRN and VTA via fMRI in major depression. In the low frequency regime of BOLD fMRI, spectral slowing occurs in both nuclei, being associated to clinical scores for DRN. Our results constitute an important step in linking altered neuronal activity in these nuclei to system-level integration patterns observed via functional connectivity assessment via fMRI.

## Author contributions

AA and AW were involved in the conception and design of the study. AW, DJ, ML, SJ, and AA developed the methods. AA, HK, ML, and SJ involved in the acquisition of the data; AW, AA, and HK performed the analyses. AW, DJ, SJ, ML, and AA were involved in the preparation of the manuscript.

### Conflict of interest statement

The authors declare that the research was conducted in the absence of any commercial or financial relationships that could be construed as a potential conflict of interest. The reviewer NY and handling Editor declared their shared affiliation.
